# Derivative-free neural network for optimizing the scoring functions associated with dynamic programming of pairwise-profile alignment

**DOI:** 10.1186/s13015-018-0123-6

**Published:** 2018-02-15

**Authors:** Kazunori D. Yamada

**Affiliations:** 1 Graduate School of Information Sciences, Tohoku University, 6-3-09, Aramaki-Aza-Aoba, Aoba-ku, Sendai, 980-8579 Japan; 2 Artificial Intelligence Research Center, National Institute of Advanced Industrial Science and Technology (AIST), Tokyo, Japan

**Keywords:** Dynamic programming, Profile alignment, Neural network, Evolutionary strategy, Derivative-free optimization

## Abstract

**Background:**

A profile-comparison method with position-specific scoring matrix (PSSM) is among the most accurate alignment methods. Currently, cosine similarity and correlation coefficients are used as scoring functions of dynamic programming to calculate similarity between PSSMs. However, it is unclear whether these functions are optimal for profile alignment methods. By definition, these functions cannot capture nonlinear relationships between profiles. Therefore, we attempted to discover a novel scoring function, which was more suitable for the profile-comparison method than existing functions, using neural networks.

**Results:**

Although neural networks required derivative-of-cost functions, the problem being addressed in this study lacked them. Therefore, we implemented a novel derivative-free neural network by combining a conventional neural network with an evolutionary strategy optimization method used as a solver. Using this novel neural network system, we optimized the scoring function to align remote sequence pairs. Our results showed that the pairwise-profile aligner using the novel scoring function significantly improved both alignment sensitivity and precision relative to aligners using existing functions.

**Conclusions:**

We developed and implemented a novel derivative-free neural network and aligner (Nepal) for optimizing sequence alignments. Nepal improved alignment quality by adapting to remote sequence alignments and increasing the expressiveness of similarity scores. Additionally, this novel scoring function can be realized using a simple matrix operation and easily incorporated into other aligners. Moreover our scoring function could potentially improve the performance of homology detection and/or multiple-sequence alignment of remote homologous sequences. The goal of the study was to provide a novel scoring function for profile alignment method and develop a novel learning system capable of addressing derivative-free problems. Our system is capable of optimizing the performance of other sophisticated methods and solving problems without derivative-of-cost functions, which do not always exist in practical problems. Our results demonstrated the usefulness of this optimization method for derivative-free problems.

## Background

The profile-comparison alignment method with a position-specific scoring matrix (PSSM) [1] is a highly accurate alignment method. The PSSM is a two dimensional vector (matrix) that stores sequence lengths, with each element in the vector consisting of a 20-dimensional numerical vector where each value represents the likelihood of the existence of each amino acid at a site in a biological sequence. Here, we designed the vector inside a PSSM as a position-specific scoring vector (PSSV). In profile alignment, cosine similarity or the correlation coefficient between two PSSVs is generally computed to measure similarity or dissimilarity between the two sites in the sequences of interest using dynamic programming (DP) [2, 3]. Profile alignment methods using these functions have long been used successfully [4], and the performance of profile alignment has improved in recent decades. As examples, HHalign improved alignment quality using profiles constructed with a hidden Markov model, which provided more information than a PSSM [5], MUSTER incorporated protein-structure information into a profile [3], and MRFalign utilized Markov random fields to improve alignment quality [6]. However, although various methods have been devised from different perspectives, studies to develop the scoring function for PSSV comparison using sophisticated technologies are lacking. Moreover, there remains room for improvement in the performance of sequence alignment, especially for remote sequence alignment [7–9]; therefore, it is important to continue developing aligners from various perspectives. Although cosine similarity or a correlation coefficient is normally used for comparison of PSSVs, in principle, they are unable to capture nonlinear relationships between vectors. However, the similarity between two amino acid positions is not always explained by linear relationship, which is merely one of a particular case of a nonlinear relationships. Because scoring functions are directly related to the quality of biological-sequence alignment, development of a novel function capable of capturing nonlinear relationships reflecting similarity between two sites in sequences is required.

The expression of nonlinear functions can be realized by neural networks. A neural network is a computing system that mimics biological nervous systems. Theoretically, if a proper activation function is set on middle layer(s) of a network, it can approximate any function including nonlinear functions [10]. Neural networks have attracted interest from various areas of research, including bioinformatics, due to recent advances in computational technologies and the explosive increase in available biological data. In recent years, these algorithms have been vigorously applied for bioinformatics purposes, including several studies associated with application of deep neural network models to predict protein–protein interactions [11, 12], protein structure [13, 14], and various other biological conditions, such as residue-contact maps, backbone angles, and solvent accessibility [15, 16]. These neural networks used backpropagation as a solver, which requires a derivative-of-cost function to search for optimal parameters [17]. However, few studies have implemented derivative-free neural networks.

Since neural networks are capable of implementing nonlinear functions, they are suitable for developing novel scoring functions for PSSV comparison. Therefore, in this study we used a neural network to optimize a nonlinear scoring function associated with PSSV comparison by combining two PSSVs as an input vector. Since we lacked a target vector normally required to implement supervised learning, we calculated the entire DP table for the input sequences, and the difference between the resultant alignment and the correct alignment was used to calculate cost of learning. Due to the nature of the problem, we could not use the backpropagation method as a solver for optimal weight and bias searches, because we lacked the derivative-of-cost function normally required. These issues are common when applying such methods to real-world problems. It is impossible to calculate a derivative for problems where the output vectors are not directly used for computation of cost function such as cross entropy or square error [18]. In this study, the outputs of a neural network were similarity score between two PSSVs and not directly used for computation of the cost function but indirectly used for computation of dynamic programming. The possibility of computing neural network inferences without derivatives would be useful for solving such problems.

Here, we used a covariance matrix adaptation-evolution strategy (CMA-ES) [19] as a solver for the neural network to implement a derivative-free neural network system. CMA-ES is an adaptive-optimization method that modifies the basic evolutionary strategy [20]. As advantages, it requires a smaller number of hyperparameters than other evolutionary strategy methods [19], and when the dimensionality of an objective function is large, it offers higher computation speeds relative to other derivative-free optimization methods, such as the Nelder–Mead method, which requires computation times proportional to the dimensionality of the objective function [21]. In this study, we implemented a derivative-free neural network system using CMA-ES and produced a high-performance scoring function for remote-sequence alignment. Our goal was to develop a novel scoring function for profile alignment method and provide a novel derivative-free learning method useful for optimizing derivative-free problems.

## Methods

### Dataset

We downloaded the non-redundant subset of SCOP40 (release 1.75) [22], in which sequence identity between any sequence pair is < 40%, from ASTRAL [23]. We selected the remote-sequence subset, because we wanted to improve remote-sequence alignment quality, which is generally a difficult problem for sequence aligners. SCOP is a protein-domain database where sequences are classified in a hierarchical manner by *class*, *fold*, *superfamily*, and *family*. To guarantee independence between a learning and test dataset, all notations of *superfamily* in the dataset were sorted in alphabetical order, and all *superfamilies*, the ordered numbers of which were multiples of three, were classified into a learning dataset, whereas the others were classified into a test dataset. This procedure is often used in existing studies for protein sequence analysis [8, 9], in order to cope with a problem of overfitting. We obtained 3726 and 6843 sequences in the learning and test datasets, respectively. We then randomly extracted a maximum of 10 pairs of sequences from each *superfamily* to negate a bias induced by different volumes of each *superfamily* and used these sequence pairs for subsequence construction of a PSSM. We confirmed that sequences in each pair were from the same *family* in order to obtain decent reference alignments. We ultimately obtained 1721 and 3195 sequence pairs in the learning and test datasets, respectively. These datasets are provided at https://github.com/yamada-kd/nepal.

### Construction of profiles and reference alignments

We constructed PSSMs for all sequences in the learning and test datasets using DELTA-BLAST version 2.2.30+ with the Conserved Domain Database for DELTA-BLAST version 3.12 [24]. Reference alignments were constructed through structural alignment of protein steric structures, which corresponded to sequences of interest using TM-align [25]. All structure data were also downloaded from ASTRAL [23].

### Learning network

**Fig. 1:**
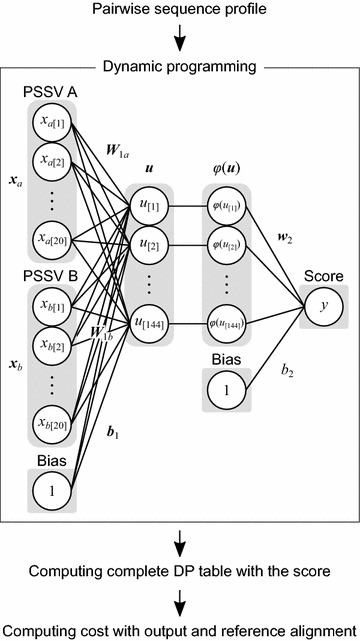
Schematic diagram of the learning network. Upper case letters in italics and bold, lowercase letters in italics and bold, and lowercase letters in italics represent matrix, vector, and scalar values, respectively. Here, ***x***_*a*_ and ***x***_*b*_ represent the input vector, ***W***_1*a*_, ***W***_1*b*_, and **w**_2_ are weight matrices and vectors, ***b***_1_ and *b*_2_ are bias vectors and scalar values, ***u*** is the middle layer vector, and *y* is the output value (the similarity score between PSSV A and PSSV B). The activating function is represented by *φ*(***u***). The square bracket represents the index of each vector

Figure 1 shows the learning network computed in this study. We calculated similarity scores between two PSSVs using the neural network. Initially, the summation of matrix products between ***x***_*a*_ (PSSV A) and ***W***_1*a*_, ***x***_*b*_ (PSSV B) and ***W***_1*b*,_ and 1 (bias) and ***b***_1_ in the neural network were calculated. Here, ***x***_*a*_ and ***x***_*b*_ were 20-element vector calculated from a DELTA-BLAST search, where each element of the vector represented the likelihood of existence of each amino acid, and ***W***_1*a*_, ***W***_1*b*,_ 1, and ***b***_1_ were weight and bias parameters of the neural network. The resultant vector was transformed by an activating function, *φ*(*u*). The rectified linear unit [26] was utilized as the activation function:

1 $$\varphi \left( u \right) = \hbox{max} \left( {0,\;u} \right).$$

The summation of the dot products between the transformed vector, *φ*(***u***) and ***w***_2_, and 1 and *b*_2_ was calculated, where ***u*** was a vector representing the middle layer, and ***w***_2_, 1, and *b*_2_ were parameters of the neural network. The resultant value was used as the similarity score for the two sites. Namely, the forward calculation was computed by the equation:

2 $$y = \varvec{w}_{2} \varphi \left( {\varvec{x}_{a} \varvec{W}_{1a} + \varvec{x}_{b} \varvec{W}_{1b} + \varvec{b}_{1} } \right) + b_{2} ,$$

where *y*, a scalar value, is the similarity score.

The complete DP table was calculated using the similarity score, and a final pairwise alignment was produced. The pairwise alignment and its corresponding reference alignment were compared to each other, and an alignment sensitivity score was calculated. Subtraction of the alignment-sensitivity score from 1 was used as the cost for searching the optimal weight using the neural network with CMA-ES.

We set the weights ***W***_1*a*_ and ***W***_1*b*_ equal to each other (shared weight) in order to apply the same value to the network outputs, even though the input order of the two PSSVs was opposite one another:

3 $$\varvec{W}_{1a} = \varvec{W}_{1b} .$$

The number of units of the middle layer was set to 144. To compute backward calculations for the network, we used CMA-ES. As hyperparameters for CMA-ES, we set σ, λ, and μ to 0.032, 70, and 35, respectively. Here, σ is almost equivalent to the step size (learning rate) of the normal gradient-descent method, and λ and μ indicate the number of descendant and survival individuals in the evolutionary process, respectively. We input training datasets into the learning system in a batch manner. The maximum number of epochs was set to a relatively small number (150) to accommodate our computational environment. During learning, the performance of the scoring function was evaluated on the validation dataset starting from the 50th epoch to the final epoch in five steps, and a scoring function that maximized the validation score was selected as the final product of the learning process. The initial weight and bias were derived from parameters that mimicked the correlation coefficient. To generate the initial weight, we randomly generated 200,000 PSSV pairs and learned them using multilayer perceptron with hyperparameters (the dimensions of the weight and activating function) identical to those already described. In addition to the parameters, we simultaneously optimized the open- and extension-gap penalties, the initial values of which were set to − 1.5 and − 0.1, respectively. The source code for our learning method is provided at https://github.com/yamada-kd/nepal.

### Alignment algorithm

In this study, we implemented the semi-global alignment method (global alignment with free-end-gaps) [27, 28].

### Metrics of alignment quality

Alignment quality was evaluated using alignment sensitivity and precision [9]. The alignment sensitivity was calculated by dividing the number of correctly aligned sites by the number of non-gapped sites in a reference alignment. By contrast, alignment precision was calculated by dividing the number of correctly aligned sites by the number of non-gapped sites in a test alignment.

### Calculation of residue interior propensity

The relative accessible surface area (rASA) for residues of all proteins in the learning and test datasets was calculated by areaimol in the CCP4 package version 6.5.0 [29]. The residues associated with rASA < 0.25 were counted as interior residues, and the other residues were counted as surface residues based on methods used previously [30]. We divided the ratio of the interior residues by the background probability associated with these residues to calculate the residue interior propensity, which represented the likelihood of a residue existing inside a protein. A propensity > 1 signified that the probability of the residue being inside the protein was higher than expected.

### Statistical analysis

Statistical tests, including Wilcoxon signed-rank test with Bonferroni correction and Spearman’s rank correlation, were computed using the functions pairwise.wilcox.test() and cor.test() from R version 2.15.3 (https://cran.r-project.org/), respectively.

## Results and discussion

### Gap optimization of existing functions

First, we conducted gap-penalty optimization of the existing scoring functions, such as cosine similarity and correlation coefficient, on the learning dataset. We computed both alignment sensitivity and precision for aligners using these functions, changing open- and extension-gap penalties by increments of 0.1 from − 2.0 to − 0.6 and from − 0.4 to − 0.1, respectively, with the best alignment sensitivity selected as the optimal combination. As shown in Table 1, the best gap-penalty combination for cosine similarity and correlation coefficient was (− 1.0, − 0.1) and (− 1.5, − 0.1), respectively.

**Table 1:** Gap optimization of the existing scoring function

	Open	Extension	Sensitivity	Precision
Cosine	− 1.0	− 0.1	0.6837	0.6550
CC	− 1.5	− 0.1	0.6882	0.6613

Open and extension indicate optimized open- and extension-gap penalties, respectively, and cosine and CC represent aligners using cosine similarity and correlation coefficient as scoring functions, respectively

### Optimization of the scoring function and gap penalties

We then optimized the scoring function on the neural network with CMA-ES. During learning, we randomly divided the learning dataset into two subsets (training and validation datasets) and observed training and validation curves to confirm overfitting did not occur. The learning and validation dataset included 1536 and 160 pairwise PSSM sets and the corresponding reference alignments as targets, respectively. Because calculation of learning using our parameter settings requires > 100,000 × DP (the size of the training dataset × λ) per epoch, the consumption of computer resources was large, and calculation time was long, even when 24 threads were used with the C++ program. Therefore, we set the maximum limit for epoch to a relatively small number (150). To maximize the learning within the finite learning time, we monitored the performance of intermediate scoring functions on the validation dataset every fifth epoch. According to the validation scores, we ultimately selected a scoring function derived from the 145th epoch, which maximized the validation score, as the final product of learning. In addition to the scoring function, open- and extension-gap penalties are also vital parameters for DP, which outputs optimal alignments against four parameters, including the pairwise sequences, a scoring function, and open- and extension-gap penalties. We optimized the gap penalties along with other parameters, and simultaneously optimized gap penalties using a scoring function to obtain final weight and bias matrices representing the substance of a novel scoring function and optimal gap-penalty combinations, respectively. Our results allowed realization of an optimal combination of open- and extension-gap penalties for the final weight and bias matrices (approximately − 1.7 and − 0.2, respectively).

We implemented a pairwise-profile aligner with the weight and bias matrices as a novel scoring function and named it Neural network Enhanced Profile Alignment Library (Nepal). Nepal accepts pairwise sequences and their corresponding PSSM as an input and outputs a pairwise alignment for the input sequences. The scoring function is performed by a neural network, and the similarity score, *y*, between two PSSVs (***x***_*a*_ and ***x***_*b*_) is calculated using Eq. 2, with three weight (***W***_1*a*_, ***W***_1*b*_, and ***w***_**2**_) and two bias (***b***_1_ and *b*_2_) matrices the final products of learning. Our aligner and scoring function (weight and bias matrices) can be downloaded from https://github.com/yamada-kd/nepal.

### Benchmarking of Nepal and other aligners using an existing function on the test dataset

We then conducted a benchmark test of Nepal and other aligners using an existing function on the test dataset. In addition to profile-comparison methods, we examined the performance of sequence-comparison aligners with different substitution matrices, such as BLOSUM62 [31] and MIQS [32], as references. We used − 10 and − 2 as open- and extension-gap penalties, respectively, based on a previous study [32]. When calculating alignment quality, the test dataset was further categorized into remote and medium subsets depending on the pairwise sequence identity of the reference alignments. The remote and medium subsets included sequence pairs where each sequence identity was not < 0 and < 20% and not < 20 and < 40%, respectively. Generally, a pairwise alignment between sequences of lower identity under the twilight zone is a more difficult problem [7].

Table 2 shows the alignment-quality scores for each method. Results showed that among the existing methods, including sequence-comparison methods, the profile-comparison method, which implemented correlation coefficient as a scoring function, performed the best. By contrast, Nepal improved both alignment sensitivity and precision relative to the profile-comparison method. We evaluated the statistical significance between all pairwise combinations of methods individually based on alignment sensitivity or precision on every dataset subset using a Wilcoxon signed rank test with Bonferroni correction. The results indicated that the improved results derived from Nepal were statistically significant (α < 0.01), suggesting that the novel derivative-free neural network succeeded in optimizing the scoring function. Comparison between sequence-based methods with different substitution matrices, such as MIQS and BLOSUM62, showed that the improvement derived from using MIQS as compared with BLOSUM62 was more significant for the remote subset than the medium subset. This result was reasonable, because MIQS was originally developed to improve remote homology alignment. This trend was also observed in the relationship between Nepal and the profile aligners using correlation coefficient. Here, Nepal improved both alignment sensitivity and precision by ~ 4 and ~ 1% in the remote and medium subsets, respectively. This indicated that the novel scoring function was optimized for remote sequence alignment rather than alignment of closer sequences. This was expected, because alignment of sequences with closer identities is easier than those with remote identities. Therefore, during optimization, the novel scoring function would be naturally optimized for remote sequence alignment. These results suggested that the learning system described in this study represented a scoring function useful for remote sequence alignment. Remote homology detection is the most important problem for sequence-similarity searches [32, 33]. The novel scoring function presented in the present study could be useful for improving the performance of existing similarity search methods.

**Table 2:** Comparison of Nepal with other alignment methods

	Remote [0,20)^a^ (1405 files)	Medium [20,40)^a^ (1790 files)	All [0,40)^a^ (3195 files)
Sensitivity			
Nepal	0.5317	0.8343	0.7012
Cosine	0.5045**	0.8246**	0.6838**
CC	0.5135**	0.8269**	0.6891**
MIQS	0.2775**	0.7316**	0.5319**
BL62	0.2333**	0.6955**	0.4923**
Precision			
Nepal	0.5031	0.8102	0.6751
Cosine	0.4753**	0.7999**	0.6571**
CC	0.4858**	0.8032**	0.6636**
MIQS	0.2654**	0.7134**	0.5164**
BL62	0.2317**	0.6902**	0.4885**

Cosine, *CC*, MIQS, and BL62, indicate profile comparison methods with cosine similarity and correlation coefficient and sequence comparison methods with MIQS and BLOSUM62

** P < 0.01, Wilcoxon signed rank test with Bonferroni correction

^a^Sequence identity (%) of each division

### Importance of attributes according to the connection-weight method

**Fig. 2:**
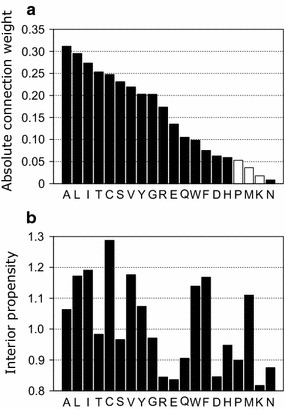
**a** Absolute connection weight for each attribute corresponding to the profile value of each amino acid. Filled and open bars represent positive and negative signs of the original connection weights, respectively. **b** The propensity for the residue to be buried within the protein

We calculated the importance of 20 attributes of input vectors using the connection-weight method [34], where absolute connection values represent the importance of each amino acid for profile alignment. As shown in Fig. 2a, the connection weights against each attribute (each amino acid) were distributed to various values, indicating that the scoring function described here adequately distinguished the importance of an attribute against other attributes, depending on the variety of amino acids.

Based on these results, the connection weights of hydrophobic residues, such as Leu, Ile, and Val, were of higher value. These residues are located mostly inside the hydrophobic cores of proteins. Additionally, as shown in Fig. 2b, other residues, which often buried within proteins, such as Ala, Cys, and Tyr, were also of higher importance. By contrast, residues often located on the protein surface, such as Asp, Pro, Lys, and Asn, were of lower importance. The Spearman’s rank correlation coefficient between the connection weight and interior propensity was ~ 0.6 (P < 0.05), meaning that the importance of attributes was related to the propensity of residues to be located on the interior of the protein. While residues located at the protein surface are subject to higher mutation rates, buried residues are less susceptible to mutation [35], because protein structure can be disrupted by mutation of residues buried in the core of the protein, which could potentially result in collapse of the hydrophobic core [36]. The scoring function presented in this study was optimized for the alignment of remote homologous sequences. According to a previous study based on substitution matrices [37], residue hydrophobicity was the dominant property of remote sequence substitution rather than simple mutability. This fact partially explains why residues occupying interior locations are considered more meaningful for remote sequence alignment. Because our scoring function was optimized for remote sequence alignment, it considered these amino acids as important attributes. This characteristic of the scoring function represents a superior attribute of our method relative to existing methods.

**Fig. 3:**
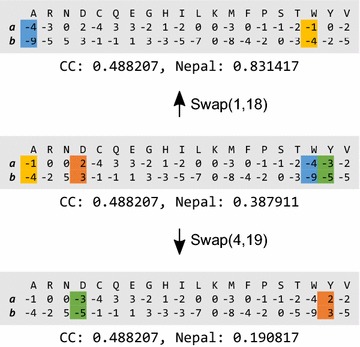
Transition of similarity scores depending on site swapping. In each panel, ***a*** and ***b*** represent PSSV A and B, respectively. The middle panel represents an original PSSV and similarity scores calculated using correlation coefficient (CC) and Nepal. The top and bottom panels show the resulting PSSVs and similarity scores

Additionally, although the connection weight consisted of various values, it contributed to increases in the expressive power of the novel scoring function. We calculated the similarity score between PSSV A (***a***) and B (***b***), resulting in 0.488207 and 0.387911 when calculated using the correlation coefficient and Nepal methods, respectively (Fig. 3, middle panel). The scores calculated using the correlation coefficient did not change when the 1st and 18th sites or the 4th and 19th sites were swapped. These results could be inappropriate, because the converted PSSV obtained after swapping was not identical to the original, which could represent a potential drawback of using unweighted linear functions, such as cosine similarity and correlation coefficient. By contrast, the Nepal scores changed after swapping and varied along with changes in the PSSV. This expressiveness represents a merit of nonlinear functions. There were ~ 290,000 overlaps following the calculation of similarity scores to six decimal places against 1 million randomly generated PSSVs using the correlation coefficient method, whereas there were ~ 180,000 overlaps when Nepal was used. These overlaps would negatively affect DP computation, because higher overlap scores would cause difficulties in determining the correct path, especially during the computation of a maximum of three values derived from different sides of DP cell. Our results showed that the use of different weights by the connection-weight method and based on amino acid variety is one reason why the Nepal scoring method improved alignment quality as compared with the existing scoring functions.

## Conclusions

In this study, we optimized a scoring function for pairwise-profile alignment using a machine-learning method mimicking a nonlinear function. Our method enabled computational optimization, regardless of whether given problem involved a derivative-of-cost function, given that this scenario is not always present in real-world problems. In this study, we developed a novel derivative-free neural network with CMA-ES and successfully applied this learning system to optimize a scoring function for pairwise-profile alignment. Nepal significantly improved the alignment quality of profile alignments, especially for alignments based on remote relationships, as compared with existing scoring functions. Moreover, Nepal improved alignment quality based on the adaptation to remote sequence alignment and the increasing expressiveness of the similarity score. This method alone is not practical as a standalone pairwise-profile aligner; however, because the novel scoring function involves a simple matrix operation using parameters provided on the website, the performance of distant homology detection or multiple-sequence-alignment methods for remote homologous sequences might be further improved by incorporation of our scoring function. Finally, the goal of the study was not only to provide an alternative alignment method but also to provide a novel learning system capable of addressing derivative-free problems. Our system will be useful for optimizing the scoring functions of other sophisticated methods such as similarity search, multiple-sequence alignment and etc.

## Abbreviations

CMA-ES: covariance matrix adaptation evolution strategy
DP: dynamic programming
PSSM: position-specific scoring matrix
PSSV: position-specific scoring vector
